# Statin use associated with a reduced risk of hip fracture in patients with gout

**DOI:** 10.1016/j.bonr.2024.101799

**Published:** 2024-08-19

**Authors:** Chun-Ming Chen, Wan-Ting Huang, Sheng-Feng Sung, Chih-Cheng Hsu, Yueh-Han Hsu

**Affiliations:** a Division of Allergy, Immunology, and Rheumatology, Department of Internal Medicine, Ditmanson Medical Foundation Chia-Yi Christian Hospital, Chiayi 60002, Taiwan; b Clinical Medicine Research Center, Ditmanson Medical Foundation Chia-Yi Christian Hospital, Chiayi 60002, Taiwan; c Division of Neurology, Department of Internal Medicine, Ditmanson Medical Foundation Chia-Yi Christian Hospital, Chiayi 60002, Taiwan; d Department of Beauty & Health Care, Min-Hwei Junior College of Health Care Management, Tainan 73658, Taiwan; e Institute of Population Health Sciences, National Health Research Institutes, Miaoli 35053, Taiwan; f Department of Family Medicine, Min-Sheng General Hospital, Taoyuan 33044, Taiwan; g Department of Internal Medicine, Ditmanson Medical Foundation Chia-Yi Christian Hospital, Chiayi 60002, Taiwan; h Department of Nursing, Min-Hwei Junior College of Health Care Management, Tainan 73658, Taiwan; i Department of Medical Research, China Medical University Hospital, Taichung 40447, Taiwan

**Keywords:** Gout, Statins, Effectiveness, Osteoporosis, Hip fracture

## Abstract

Studies show that statins users are at reduced risk of fracture and improved bone mineral density. However, the clinical effectiveness of statin use in patients with gout has not been investigated. This retrospective cohort study used data from Taiwan's National Health Insurance Research Database, consisting of 3443 patients with gout using statins aged 50 years and above and 6886 gout patients of non-statin users matched by sex, age and propensity score. The Cox proportional hazards regression analysis showed that statin use was associated with a reduced risk of hip fracture (adjusted hazard ratio [aHR] = 0.78, 95 % confidence interval [CI] = 0.64–0.94) after controlling for potential confounding factors. The association was significant in both genders aged 50–64 years, with aHRs of near 0.35, but not in the elderly. In addition, women aged 50–64 years who used statins also exhibited a lower risk of vertebral fracture (aHR = 0.70, 95 % CI = 0.50–0.99), but not men. In conclusion, the stating use in gout patients could reduce fracture risk for younger patients. Further research is warranted to confirm these findings.

## Highlights

• Statin therapy links with reduced hip fracture risk in patients with gout over the age of 50.
• The association is significant in 50–64 years old men and women.
• Women in this age using statins also had a lower vertebral fracture risk, but not men.

## 1 Introduction

Osteoporosis is a systemic skeletal disease characterized by low bone mass and microarchitectural deterioration of bone tissue. Consequently, osteoporosis causes bones to become more fragile and prone to fractures (Compston et al., 2019). In Taiwan, the epidemiology data showed that the prevalence of osteoporosis increased from 17.4 % in 2001 to 25.0 % in 2011 (Tai et al., 2023). Consequently, the hip fracture cases had a 49.9 % increase (14,978 versus 22,465 cases) in this period (Tai et al., 2023). Fractures often lead to serious consequences, including high medical cost and mortality. Vertebral fracture can temporarily increase the risk of mortality by two to eight times, and 36 % of individuals with hip fracture may die within a year (Schousboe, 2016; Bhandari and Swiontkowski, 2017). The economic burden of fragility fractures is substantial. A Singapore study has estimated that there will be a 57.9 % increase of incident osteoporotic fractures between 2017 and 2035 with a 1.6-fold increase for the corresponding fracture-related costs (Chandran et al., 2019).

Gout, caused by monosodium urate crystal deposition in joints and tissues, is the most common inflammatory arthritis, with a prevalence of 3 % to 6 % in men and 1 % to 3 % in women (Clebak et al., 2020). Gout is also common in Taiwan, with an estimated prevalence rate of 6.24 % (Kuo et al., 2015). Although findings regarding gout and osteoporotic fractures have been inconsistent (Tzeng et al., 2016; Kim et al., 2017), a meta-analysis revealed that gout is associated with an increased risk of fracture, including osteoporotic fracture (Zong et al., 2019).

According to a report from the Third National Health and Nutrition Examination Survey in the United States, the prevalence of metabolic syndrome is remarkably high among patients with gout (Choi et al., 2007). A recent study in Taiwan also found patients with gout are at an adjusted hazard ratio (aHR) of 2.55 for developing hyperlipidemia, compared to individuals without gout (Fang et al., 2020). Statins have been demonstrated to be effective in managing hyperlipidemia and are extensively employed for preventing cardiovascular disorders (Michos et al., 2019). They may have beneficial effects with respect to increasing bone mineral density (BMD) because the pleiotropic effects on bone metabolism reduce bone resorption and promote bone formation (Gonyeau, 2005). Statin therapy is thus reported to be effective in reducing the risk of fracture and improving BMD at the lumbar spine and total hip (An et al., 2017). Given their role in dyslipidemia management, statins are commonly prescribed to patients with gout. However, to date, the potential benefit of statin use in reducing the risk of osteoporosis and bone fractures in patients with gout remains unclear. Hence, this study assessed the association between statin therapy and the risk of osteoporosis and fractures among patients with gout.

## 2 Material and methods

### 2.1 Data sources

The data analyzed in this study were retrospectively obtained from a subset of the National Health Insurance Research Database (NHIRD), that is, from the Longitudinal Health Insurance Database 2005 (LHID2005), which is managed by Taiwan's National Health Research Institutes. Taiwan's National Health Insurance program began operating in 1995; it currently covers 99.5 % of the residents of Taiwan. The LHID2005 contains the data of 2 million insured population randomly selected from the 2005 registry of the NHIRD; it contains all claims data collected on these individuals from January 1, 2000, to December 31, 2018.

In this study, data from 2002 to 2012 were included to ensure that every patient's medical history could be traced for at least 2 years. Researchers can access the LHID2005 upon receiving approval. It contains scrambled patient identification numbers, date of birth, sex, diagnostic codes (based on the *International Classification of Diseases, Ninth Revision, Clinical Modification* [*ICD-9-CM*] codes), and prescription drug information, among other basic data. *This study was approved by t*he Research Ethics Committee of Ditmanson Medical Foundation Chia-Yi Christian Hospital (*Approval No.* CYCH-IRB-2021015).

### 2.2 Study population

**Fig. 1:**
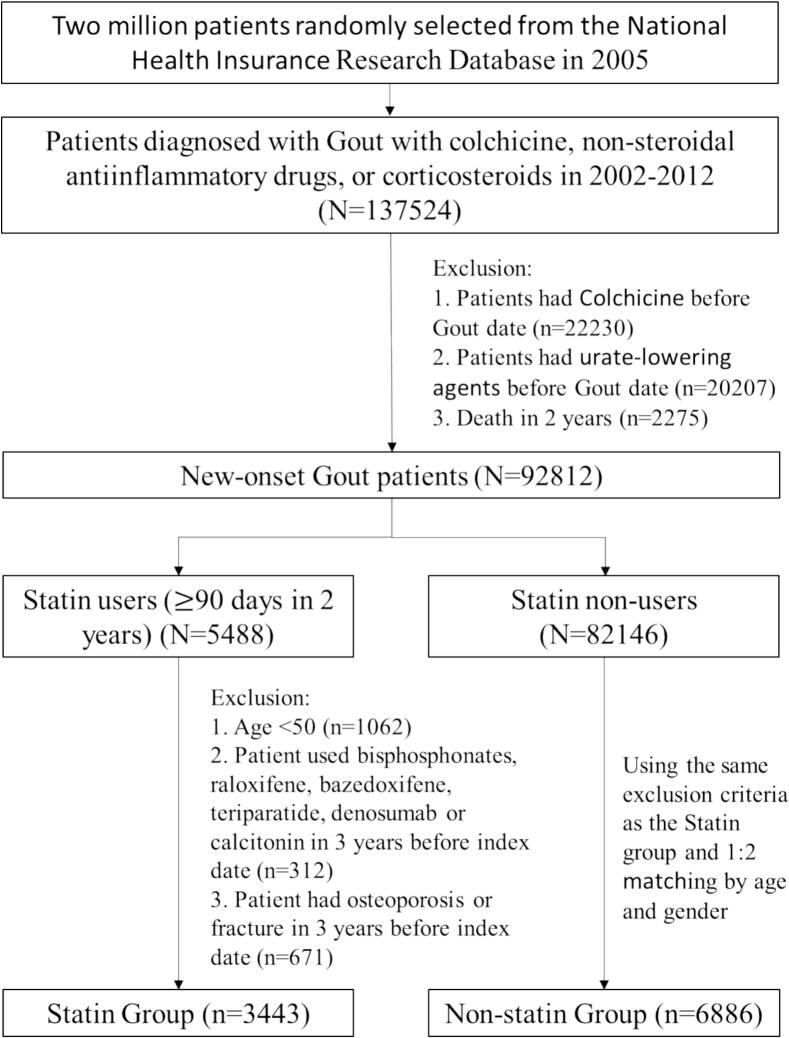
Flow chart showing selection of study participants.

This population-based cohort study assessed the association between the use of statin and osteoporotic fractures in patients with gout. The study included patients who were given a diagnosis of gout (*ICD-9-CM*: 274) and were prescribed colchicine, nonsteroidal anti-inflammatory drugs (NSAIDs), or corticosteroids during the same visits in 2002–2012. To ensure that the patients had a new diagnosis of gout, individuals who had a previous diagnosis of gout were excluded. Patients who received colchicine or urate-lowering agents before receiving a diagnosis of gout were also excluded. The statin group included patients with gout who had used statins for >90 days within 2 years after the date of diagnosis, with the 91st day being counted as the index date. The non-statin group included patients with gout who did not use any statins within 2 years after the date of diagnosis. Patients were included in the non-statin group by using 1:2 matching based on age and sex. Patients with incomplete insurance claims data and those younger than 50 years were excluded from the study. Patients who received bisphosphonates, raloxifene, bazedoxifene, teriparatide, denosumab, or calcitonin before the index date or those who had a history of osteoporosis or fractures within 3 years before the index date were also excluded. (Fig. 1).

### 2.3 Outcomes

The primary outcome was a diagnosis of osteoporosis (*ICD-9-CM*: 733.0) and any type of fracture (*ICD-9-CM*: 805–829; *International Classification of Diseases, Tenth Revision, Clinical Modification* [*ICD-10-CM*]: S02, S12, S22, S32, S42, S52, S62, S72, S82, S92, M484, and M485). All individuals were followed from the index date to the development of the primary outcome or December 31, 2018 (the last date in our study database). In addition to the primary outcome, three subgroups of fractures that are closely related to osteoporosis were analyzed: hip fracture (*ICD-9-CM*: 820; *ICD-10-CM*: S72), vertebral fracture (*ICD-9-CM*: 805; *ICD-10-CM*: S22), and upper limb fracture (*ICD-9-CM*: 814.0×, 814.1×, 813.4×, 813.5×, 812.0×, and 812.1×; *ICD-10-CM*: S42 and S52).

### 2.4 Comorbidities and medications

This study included comorbidities and medications as potential confounding factors. The most frequently identified contributing comorbid conditions were alcohol-related disorder (*ICD-9-CM*: 291, 303, 305, 571.0, 571.1, 571.2, 571.3, 790.3, and V11.3), coronary artery disease (*ICD-9-CM*: 411–414), chronic obstructive pulmonary disease (COPD; *ICD-9-CM*: 490–496), diabetes mellitus (*ICD-9-CM*: 250), end-stage renal disease (ESRD, *ICD-9-CM*: 585 from catastrophic illness files), hypertension (*ICD-9-CM*: 401–405), Parkinson's disease (*ICD-9-CM*: 332), stroke (*ICD-9-CM*: 430–434 and 436–437), and rheumatoid arthritis (*ICD-9-CM*: 714 from catastrophic illness files). Baseline comorbidities were defined as comorbidities diagnosed more than two times before the index date. Medication history was defined on the basis of a patient having received a drug for at least 30 days within the year before the index date. The considered medications were benzodiazepines and related drugs (including zopiclone, zolpidem, zaleplon, and eszopiclone), glucocorticoids, loop diuretics, and thiazide diuretics.

### 2.5 Statistical analyses

Categorical variables are expressed as frequencies (percentages), and continuous variables are expressed as means and standard deviations. Parametric continuous data between the statin and non-statin groups were compared using *t-*tests, and categorical data were compared using chi-squared tests. The statin users to non-users hazard ratios (HR) of osteoporosis and fractures were evaluated using multivariate Cox proportional hazards regression analysis. The adjusted hazard ratios (aHR) were estimated after adjustment for potential confounders, such as age, sex, comorbidities, and medications. All statistical analyses were conducted using SAS software version 9.4 (SAS Institute Inc., Cary, NC, USA), and a two-tailed *P* value of <0.05 was considered to denote statistical significance.

## 3 Results

This study included 3443 patients in the statin group and 6886 patients with the same age (53.9 years) and sex ratio (50.8 % men) in the non-statin group (Table 1). No differences were noted in the prevalence of alcohol-related disorder, ESRD, Parkinson's disease, or rheumatoid arthritis between the statin and non-statin groups. The statin group had a significantly higher prevalence of coronary artery disease (43.5 % versus 22.3 %, *P* < 0.001), COPD (36.3 % versus 30.6 %, P < 0.001), diabetes mellitus (46.8 % versus 19.6 %, P < 0.001), hypertension (79.3 % versus 51.9 %, P < 0.001), and stroke (21.4 % versus 10.3 %, P < 0.001) than the non-statin group did. Regarding medications, the statin group had higher usage of benzodiazepines and related drugs (31.2 % versus 19.1 %, P < 0.001), glucocorticoids (8.77 % versus 7.16 %, *P* = 0.004), loop diuretics (7.90 % versus 3.47 %, P < 0.001), and thiazide diuretics (6.65 % versus 3.62 %, P < 0.001) than the non-statin group did.

**Table 1:** Demographic factors, comorbidities, and medications of patients with gout based on the usage of statin.

	Total	non use	Statin use	*P* value
	*N* = 10,329	*n* = 6886	*n* = 3443	
Age				
50–64, n(%)	5566(53.9)	3712(53.9)	1854(53.9)	0.956
≥65, n(%)	4763(46.1)	3174(46.1)	1589(46.2)	
Mean(SD)	64.82(9.43)	64.82(9.43)	64.82(9.43)	0.999
Sex				
Male, n(%)	5250(50.8)	3500(50.8)	1750(50.8)	>0.999
Female, n(%)	5079(49.2)	3386(49.2)	1693(49.2)	
Comorbidities				
Alcohol-related disorder, n(%)	198(1.92)	125(1.82)	73(2.12)	0.287
Coronary artery disease, n(%)	3033(29.4)	1536(22.3)	1497(43.5)	<0.001
COPD, n(%)	3354(32.5)	2104(30.6)	1250(36.3)	<0.001
Diabetes mellitus, n(%)	2921(28.3)	1311(19.0)	1610(46.8)	<0.001
ESRD, n(%)	55(0.53)	32(0.46)	23(0.67)	0.181
Hypertension, n(%)	6304(61.0)	3573(51.9)	2731(79.3)	<0.001
Parkinson's disease, n(%)	170(1.65)	108(1.57)	62(1.80)	0.382
Stroke, n(%)	1448(14.0)	710(10.3)	738(21.4)	<0.001
Rheumatoid arthritis, n(%)	625(6.05)	411(5.97)	214(6.22)	0.620
Medications				
Benzodiazepines and related drugs, n(%)	2389(23.1)	1316(19.1)	1073(31.2)	<0.001
Glucocorticoids, n(%)	795(7.70)	493(7.16)	302(8.77)	0.004
Loop diuretics, n(%)	511(4.95)	239(3.47)	272(7.90)	<0.001
Thiazide diuretics, n(%)	478(4.63)	249(3.62)	229(6.65)	<0.001

Abbreviations: SD, standard deviation; COPD, chronic obstructive pulmonary disease; ESRD, end stage renal disease.

Table 2 presents the risks of osteoporosis and fractures in the statin and non-statin groups. After adjustment for age, sex, comorbidities, and medication use, the statin group exhibited a 22 % lower risk of hip fracture than the non-statin group did (aHR = 0.78, 95 % CI = 0.64–0.94). However, no significant differences were noted between the two groups for all types of fractures, vertebral fracture, upper limb fracture, and osteoporosis.

**Table 2:** Incidence rates and hazard ratios of outcomes of interest for the effect of statin.

Variables	Non-Statin			Statin			Crude HR	*P* value	Adjusted HR&	*P* value
	Event	PYs	Rate#	Event	PYs	Rate#	(95 % CI)		(95 % CI)	
All fractures	1324	63,212	2.09	657	31,621	2.08	0.99(0.90,1.09)	0.867	0.95(0.86,1.05)	0.285
Hip fracture	371	69,437	0.53	173	34,752	0.50	0.93(0.78,1.12)	0.443	0.78(0.64,0.94)	0.010
Vertebral fracture	528	68,489	0.77	256	34,311	0.75	0.97(0.83,1.12)	0.663	0.93(0.79,1.10)	0.394
Upper limb fracture	518	68,592	0.76	247	34,316	0.72	0.95(0.82,1.11)	0.537	1.01(0.85,1.19)	0.948
Osteoporosis	1360	62,735	2.17	664	31,442	2.11	0.98(0.89,1.07)	0.588	0.93(0.84,1.03)	0.149

Abbreviations: PYs, person-years; HR, hazard ratio; CI, confidence interval.

# Rate, incidence rate in per 100 person-years.

& Adjusted for Age, Sex, Comorbidities, and Medications.

Table 3 presents the incident fractures in men stratified by age group. Men who used statins had a reduced risk of hip fracture (aHR = 0.69, 95 % CI = 0.48–0.98). However, regardless of age, no differences were noted in the risks of all types of fractures, vertebral fracture, upper limb fracture, and osteoporosis between the statin and non-statin groups. Regarding hip fracture, no significant differences were noted between the men aged ≥65 years in both groups. However, the men aged 50–64 years in the statin group had a lower risk of hip fracture (aHR = 0.34, 95 % CI = 0.16–0.76) than did those in the non-statin group.

**Table 3:** Incidence rates and hazard ratios of outcomes of interest for the effect of statin in male patients, stratified by age group.

Male	Non-Statin			Statin			Crude HR	*P* value	Adjusted HR&	*P* value
	Event	PYs	Rate#	Event	PYs	Rate#	(95 % CI)		(95 % CI)	
Total, *n* = 5250										
All fractures	372	34,429	1.08	181	17,194	1.05	0.98(0.82,1.16)	0.779	0.95(0.78,1.15)	0.566
Hip fracture	125	35,935	0.35	48	18,015	0.27	0.77(0.55,1.07)	0.116	0.69(0.48,0.98)	0.037
Vertebral fracture	171	35,772	0.48	79	17,885	0.44	0.92(0.71,1.21)	0.563	0.99(0.74,1.32)	0.933
Upper limb fracture	131	35,902	0.36	64	17,932	0.36	0.98(0.73,1.32)	0.886	0.96(0.69,1.34)	0.791
Osteoporosis	377	34,259	1.10	181	17,110	1.06	0.96(0.81,1.15)	0.669	0.93(0.77,1.13)	0.477
50–64, *n* = 3087										
All fractures	153	20,763	0.74	82	10,316	0.79	1.08(0.83,1.41)	0.578	1.00(0.74,1.36)	0.996
Hip fracture	42	21,387	0.20	9	10,753	0.08	0.43(0.21,0.88)	0.020	0.34(0.16,0.76)	0.008
Vertebral fracture	64	21,310	0.30	31	10,665	0.29	0.97(0.63,1.49)	0.882	0.97(0.59,1.59)	0.910
Upper limb fracture	83	21,153	0.39	47	10,536	0.45	1.14(0.80,1.63)	0.481	1.09(0.72,1.65)	0.672
Osteoporosis	153	20,701	0.74	81	10,294	0.79	1.07(0.81,1.40)	0.644	1.02(0.75,1.39)	0.909
≥65, *n* = 2162										
All fractures	219	13,666	1.60	99	6878	1.44	0.90(0.71,1.14)	0.377	0.90(0.70,1.16)	0.426
Hip fracture	83	14,548	0.57	39	7262	0.54	0.94(0.64,1.38)	0.754	0.85(0.57,1.28)	0.436
Vertebral fracture	107	14,462	0.74	48	7220	0.66	0.90(0.64,1.26)	0.538	1.00(0.70,1.45)	0.987
Upper limb fracture	48	14,749	0.33	17	7396	0.23	0.71(0.41,1.23)	0.218	0.69(0.38,1.24)	0.214
Osteoporosis	224	13,558	1.65	100	6816	1.47	0.89(0.70,1.12)	0.322	0.88(0.68,1.13)	0.321

Abbreviations: PYs, person-years; HR, hazard ratio; CI, confidence interval.

# Rate, incidence rate in per 100 person-years.

& Adjusted for Age, Sex, Comorbidities, and Medications.

Table 4 presents findings in women stratified by age group. Regardless of age, no differences were noted in the risks of all types of fractures, upper limb fracture, or osteoporosis between the two groups. Regarding hip fracture and vertebral fracture, no differences were noted between the women aged ≥65 years in the statin and non-statin groups. However, women aged 50–64 years in the statin group had lower risks of hip fracture (aHR = 0.37, 95 % CI = 0.21–0.65) and vertebral fracture (aHR = 0.70, 95 % CI = 0.50–0.99) than did those in the non-statin group.

**Table 4:** Incidence rates and hazard ratios of outcomes of interest for the effect of statin in female patients, stratified by age group.

Female	Non-Statin			Statin			Crude HR	*P* value	Adjusted HR&	*P* value
	Event	PYs	Rate#	Event	PYs	Rate#	(95 % CI)		(95 % CI)	
Total, *n* = 5079										
All fractures	952	28,783	3.31	715	14,427	4.96	1.00(0.89,1.11)	0.968	0.95(0.85,1.07)	0.423
Hip fracture	246	33,502	0.73	125	16,737	0.75	1.02(0.82,1.26)	0.873	0.82(0.66,1.04)	0.098
Vertebral fracture	357	32,718	1.09	177	16,426	1.08	0.99(0.82,1.18)	0.882	0.91(0.75,1.10)	0.328
Upper limb fracture	387	32,690	1.18	183	16,383	1.12	0.94(0.79,1.13)	0.520	1.02(0.85,1.23)	0.835
Osteoporosis	983	28,476	3.45	483	14,332	3.37	0.98(0.88,1.09)	0.676	0.93(0.83,1.05)	0.247
50–64, *n* = 2479										
All fractures	362	14,770	2.45	193	7253	2.66	1.09(0.91,1.30)	0.347	0.99(0.82,1.21)	0.942
Hip fracture	61	16,571	0.37	18	8309	0.22	0.59(0.35,0.99)	0.048	0.37(0.21,0.65)	<0.001
Vertebral fracture	123	16,225	0.76	59	8129	0.73	0.96(0.70,1.30)	0.777	0.70(0.50,0.99)	0.046
Upper limb fracture	158	16,017	0.99	66	8065	0.82	0.83(0.62,1.10)	0.200	0.88(0.64,1.20)	0.420
Osteoporosis	367	14,676	2.50	192	7238	2.65	1.06(0.89,1.27)	0.498	0.97(0.80,1.18)	0.744
≥65, *n* = 2600										
All fractures	590	14,013	4.21	283	7175	3.94	0.94(0.81,1.08)	0.359	0.92(0.79,1.07)	0.270
Hip fracture	185	16,930	1.09	107	8428	1.27	1.16(0.92,1.48)	0.212	0.97(0.76,1.25)	0.832
Vertebral fracture	234	16,492	1.42	118	8297	1.42	1.00(0.80,1.25)	>0.999	1.00(0.79,1.26)	0.965
Upper limb fracture	229	16,673	1.37	117	8318	1.41	1.03(0.82,1.28)	0.827	1.10(0.87,1.39)	0.427
Osteoporosis	616	13,800	4.46	291	7093	4.10	0.92(0.80,1.06)	0.239	0.90(0.78,1.05)	0.176

Abbreviations: PYs, person-years; HR, hazard ratio; CI, confidence interval.

# Rate, incidence rate in per 100 person-years.

& Adjusted for Age, Sex, Comorbidities, and Medications.

## 4 Discussion

This study reveals that the use of statins by patients with gout, particularly those aged 50–64 years, can reduce the risk of hip fracture. In the analysis stratified by sex, both men and women aged 50–64 years had a lower risk of hip fracture. Women aged 50–64 years also had a lower risk of vertebral fracture.

Statins have dual beneficial effects on bone metabolism. They reduce lipid levels in osteoclasts, thereby inhibiting osteoclast formation. Moreover, they directly affect bone cells by inhibiting osteoclastogenesis, preventing osteoblast apoptosis, and promoting osteogenesis (Park-Min, 2019; Kim et al., 2021b). Through these potential mechanisms, statins can elevate BMD and reduce the risk of fractures.

The association between gout and an increased risk of fractures can be explained by two potential reasons. First, monosodium urate crystals induce inflammation, which contributes to bone loss (Polzer et al., 2010; So and Martinon, 2017). Second, gout attacks can cause difficulty in moving the joints, increasing the risk of accidents such as falls or missteps (Ou et al., 2020). Monosodium urate and cholesterol can form pathological crystals that activate the two-signal pathway, triggering inflammatory responses and the release of cytokines (So and Martinon, 2017; Koushki et al., 2021). Statins inhibit ligand-receptor binding, signal transduction, inflammatory cytokine production, and cholesterol crystal–induced inflammation in atherosclerosis (Bahrami et al., 2018; Koushki et al., 2021). The use of statins may also inhibit inflammation caused by monosodium urate crystals and then decrease bone loss. Furthermore, certain statins, such as atorvastatin and simvastatin, can lower the serum uric acid level (Derosa et al., 2016). The use of statins at higher cumulative doses or for a longer duration is associated with a reduced risk of developing gout (Lin et al., 2023). This can subsequently reduce the occurrence of accidents caused by gout attacks.

The results of previous researches on the association between statin use and osteoporotic fractures in the general population have been discrepant. A nested case–control cohort study using a Korean database revealed that statin therapy benefits vertebral fractures, but not hip fractures (Kim et al., 2021a). However, a meta-analysis examined 33 clinical trial data and concluded that statin treatment reduced risk of overall fractures for 19 % and hip fracture for 25 % (An et al., 2017). The effectiveness of statin therapy in reducing hip fracture for patients with gout in our study is alike to that found in the meta-analysis.

Studies investigating the association between statins and osteoporotic fractures focusing on patients with specific comorbidities have reported different findings. In patients with stroke, the use of statins has been associated with lower risks of osteoporosis, hip fracture, and vertebral fracture (Lin et al., 2018). No association was reported between statin use and osteoporotic fractures in patients with COPD (Chen et al., 2020). These variations in the literature might reflect the potent pleiotropic effects of statins.

The present study noted a lower risk of hip fracture in both men and women aged 50–64 years, and a lower incidence of vertebral fracture in women of the same age group, but not in men. The elderly-onset gout occurring in individuals aged 65 years or older, is characterized by a subacute or chronic beginning, with few inflammatory symptoms and frequent involvement of the small joints in the hands (De Leonardis et al., 2007). Bone loss resulting from monosodium urate crystal–induced inflammation may be lesser in patients with elderly-onset gout. This proposition was supported by another study, which revealed a modestly increased risk of incident osteoporosis in <60-year-old patients with gout during a long-term follow-up (Kwon et al., 2022). Whereas trauma is the most frequent cause of fractures at a younger age, bone fragility is a common cause of fractures at an older age (Wu et al., 2022). Vertebral fracture often occur without a fall, and most nonvertebral fractures are the consequence of falls (Tsuda, 2017). In Taiwan, hip fracture is more common in men than in women aged between 45 and 64 years (26.9/10,000 versus 20.0/10,000). However, among individuals aged 65 years or older, the prevalence is lower in men (129.7/10,000 versus 224.5/10,000) (Yang et al., 2010). This indicates that trauma has a greater impact on hip fracture than on bone fragility in individuals aged 50–64 years. Our study predicted that statin use would reduce the number of gout attacks (Lin et al., 2023), subsequently reducing the incidence of disability-related trauma and ultimately reducing the incidence of hip fracture among individuals aged 50–64 years.

The women aged 50–64 years who used statins experienced fewer vertebral fracture, although the significance of this effect was marginal. The Nutrition and Health Survey in Taiwan, performed from 2017 to 2020, revealed that the BMD of women is lower than that of men. Moreover, the difference in BMD between men and women is greater in the vertebrae than in the hip (Pan, 2022). As mentioned previously, the effectiveness of statins in reducing bone loss resulting from monosodium urate crystal–induced inflammation is more pronounced in individuals aged 50–64 years. Compared with men of this age, women aged 50–64 years can benefit more from a reduced risk of bone loss. This may explain our findings.

Gout is linked to cardiometabolic comorbidities, including hypertension, ischemic heart disease, congestive heart failure, and hyperlipidemia (Sandoval-Plata et al., 2021). From 2017 to 2020, hyperlipidemia prevalence in Taiwan was 28.2 % in men and 22.1 % in women (Pan, 2022), likely higher in gout patients. Statin therapy initiation reduces mortality risk in gout patients, especially those without pre-existing circulatory disease (Keller et al., 2018). However, only 54 % of Taiwanese patients with atherosclerotic cardiovascular disease achieve target LDL-C levels (Huang et al., 2022), with good medication adherence being key to reaching these targets (Lee et al., 2013). Our findings reveal that statin therapy has added benefits for patients with osteoporotic fracture. These findings may increase patient motivation to use statins and improve medication adherence in patients with gout.

The strengths of our study include its use of a representative, population-based database, and a follow-up period of longer than 10 years. Furthermore, our study design involved an unbiased participant selection process. Because enrollment in National Health Insurance is compulsory and all individuals in Taiwan have access to affordable health care, the likelihood of referral bias is low and the likelihood of follow-up compliance is high.

This study has some limitations. First, we identified all gout cases from claims data in the NHIRD using ICD codes, with the diagnoses solely relied on physician reports. To enhance the validity of gout diagnosis, we applied the criterion that an outpatient visit must coincide with a prescription of colchicine, NSAIDs, or corticosteroids. Furthermore, the accuracy of the diagnostic codes for fractures employed in National Health Insurance claims was previously validated for both inpatients and outpatients (Yang et al., 2010). Second, the NHIRD does not contain information on the risk factors for osteoporotic fractures, including body mass index and lifestyles such as, smoking status, alcohol consumption levels and, physical activity levels, and family history. These factors could have confounded the observed study outcomes. However, these unmeasured factors may not explain the statin therapy effectiveness (McCandless, 2013). The healthy user effect may play a role, where statin patients engage in other health-promoting behaviors. Third, because the claims data used in our study were de-identified, we were unable to include specific medical information regarding the fractures, such as the mechanism of injury. Consequently, we were unable to determine whether a fracture event was caused by a low-trauma injury. Fourth, because the sample size was limited, we considered statin use in general rather than comparing the effects of different types of statins.

## 5 Conclusion

Our study revealed that the use of statins in patients with gout is associated with a reduced risk of hip fracture. Notably, this association is significant in both genders aged 50–64 years, but not in the elderly. Women aged 50–64 years also had a lower risk of vertebral fractures, but not men.

## CRediT authorship contribution statement

**Chun-Ming Chen:** Writing – original draft, Investigation, Data curation, Conceptualization. **Wan-Ting Huang:** Methodology, Formal analysis, Data curation. **Sheng-Feng Sung:** Formal analysis, Data curation. **Chih-Cheng Hsu:** Visualization, Methodology, Investigation. **Yueh-Han Hsu:** Writing – review & editing, Supervision, Software, Formal analysis, Data curation.

## Declaration of competing interest

Chun-Ming Chen, Wan-Ting Huang, Sheng-Feng Sung, Chih-Cheng Hsu, and Yueh-Han Hsu declare that they have no conflict of interest.

## Data Availability

Data will be made available on request.
